# Gallstones Passed by an Infant

**Published:** 1882-07

**Authors:** 


### GALLSTONES PASSED BY AN INFANT.

Dr. A. D. Walker reports in the *British Medical Journal* a case of a male child, three months old, brought up at the breast, and, with exception of slight jaundice during the first month, entirely healthy, who was suddenly seized with a crying-spell which lasted six hours, requiring sedatives to quiet it. Castor oil was afterward given, which produced a free evacuation from the bowels; in this was found three gallstones. The largest one weighed two grains.—*Louisville Med. News.*

